# Supplementary material for: Stratospheric aerosol injection can weaken the carbon dioxide greenhouse effect
Source: Commun Earth Environ. 2025 Jun 20;6(1):485. doi: 10.1038/s43247-025-02466-z (PMC12181077; doi:10.1038/s43247-025-02466-z)
Supplement: Supplementary file 1 — Supplementary Information [file 43247_2025_2466_MOESM1_ESM.pdf]

Supplementary Information for

Stratospheric aerosol injection can weaken the carbon dioxide greenhouse effect

Haozhe He, Brian J. Soden, Gabriel A. Vecchi, Wenchang Yang

Correspondence to: Haozhe He, haozhe.he@princeton.edu

This PDF file includes:

Supplementary Tables 1 and 2
Supplementary Figures 1 to 12
Supplementary References 1 and 2

Supplementary Table 1. A list of experiments conducted in this study by the GFDL-AM2.5 and GFDL-CM2.5-FLOR family.

Model	Experiment name	Experiment description
AM2.5	CTL1990s	10-year atmosphere-only integration with sea surface temperatures and sea ice concentrations fixed at the 1990s climatology as well as the forcing conditions of 1990.
	CTL1990s-0p5TgStratBC	Same as CTL1990s, except that 0.5 Tg[*] BC aerosols are horizontal-uniformly prescribed at each of the highest 7 sigma levels of aerosol inputs[#], respectively.
	CTL1990s-0p5TgStratSO4	Same as CTL1990s, except that 0.5 Tg SO_4 aerosols are horizontal-uniformly prescribed at each of the highest 7 sigma levels of aerosol inputs, respectively.
	CTL1990s-5TgStratSO4	Same as CTL1990s, except that 5.0 Tg SO_4 aerosols are horizontal-uniformly prescribed at each of the highest 7 sigma levels of aerosol inputs, respectively.
FLOR	CTL1860	Coupled simulations with non-evolving pre-industrial conditions. Conditions chosen to be representative of the period prior to the onset of large-scale industrialization, with 1860 being the reference year.
	CTL1860-0p5TgStratBC	150-year coupled integration with 0.5 Tg BC aerosols are horizontal-uniformly prescribed at the second highest sigma level of aerosol inputs.
	CTL1860-5TgStratSO4	150-year coupled integration with 5.0 Tg SO_4 aerosols are horizontal-uniformly prescribed at the second highest sigma level of aerosol inputs.

[*] One teragram (Tg) is equal to one million metric tons (t).
[#] Identical aerosol inputs are used for the two model families.

Supplementary Table 2. An identical list of experiments conducted in this study by the GFDL-AM2.1 and GFDL-CM2.1 family.

Model	Experiment name	Experiment description
**AM2.1**	CTL1990s	Same as the CTL1990s of AM2.5 in Supplementary Table 1, except for 30-year integration.
	CTL1990s-0p5TgStratBC	Same as the CTL1990s-0p5TgStratBC of AM2.5, except for 30-year integrations.
	CTL1990s-5TgStratSO4	Same as the CTL1990s-5TgStratSO$_4$ of AM2.5, except for 30-year integrations.
**CM2.1**	CTL1860	Same as the CTL1860 of FLOR in Supplementary Table 1.
	CTL1860-0p5TgStratBC	Same as the CTL1860-0p5TgStratBC of FLOR, except there are 3 ensemble members branching from year 11, 101, 201 of the CTL1860 simulation.
	CTL1860-5TgStratSO4	Same as the CTL1860-5TgStratSO$_4$ of FLOR, except there are 3 ensemble members branching from year 11, 101, 201 of the CTL1860 simulation.
	1pct2×CO2	3 ensemble members of 150-year coupled integrations branching from the CTL1860 simulation, with CO_2 concentrations increasing steadily at 1% per year until doubling at year 70, after which the CO_2 concentration remains fixed for the remainder of the simulations.
	nonEQ2×-0p5TgStratBC	Similar to CTL1860-0p5TgStratBC, except these simulations branch from year 100 of three separate ensemble members of the 1pct2×CO2 simulations.
	nonEQ2×-5TgStratSO4	Similar to CTL1860-5TgStratSO4, except these simulations branch from year 100 of three separate ensemble members of the 1pct2×CO2 simulations.
	EQ2×CO2	A 200-year coupled integration branching from existing long-run and equilibrium abrupt doubling CO_2 simulations, with the aerosol file replaced by the one used in AM2.5 and FLOR models. This replacement introduces only minor impacts during the first few years, which are neglected in the analysis.
	EQ2×-0p5TgStratBC	Similar to CTL1860-0p5TgStratBC, except these simulations branch from three distinct initial conditions of the EQ2×CO2 simulations.
	EQ2×-5TgStratSO4	Similar to CTL1860-5TgStratSO4, except these simulations branch from three distinct initial conditions of the EQ2×CO2 simulations.

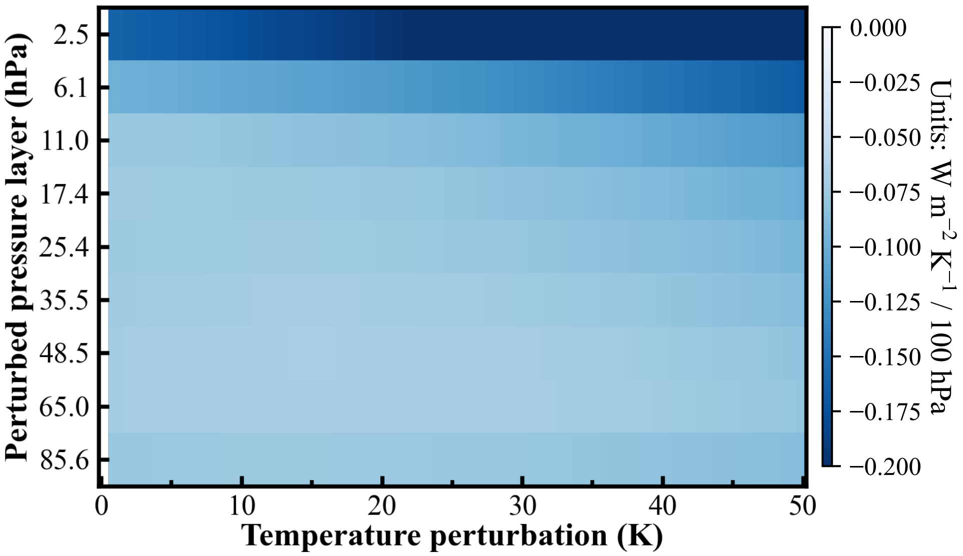

Supplementary Figure 1. Same as Fig. 1, except the sensitivity calculations are performed with the same atmospheric conditions but without CO_2.

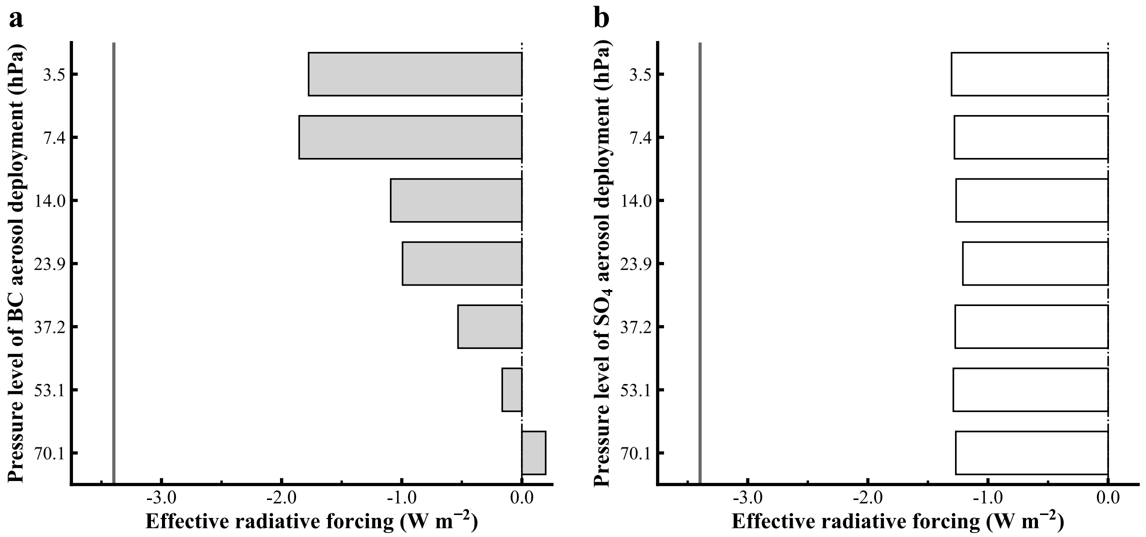

Supplementary Figure 2. Same as Fig. 2, except for the GFDL-AM2.1 model and without the effective radiative forcing results from 0.5 Tg SO_4 aerosols in (**b**).

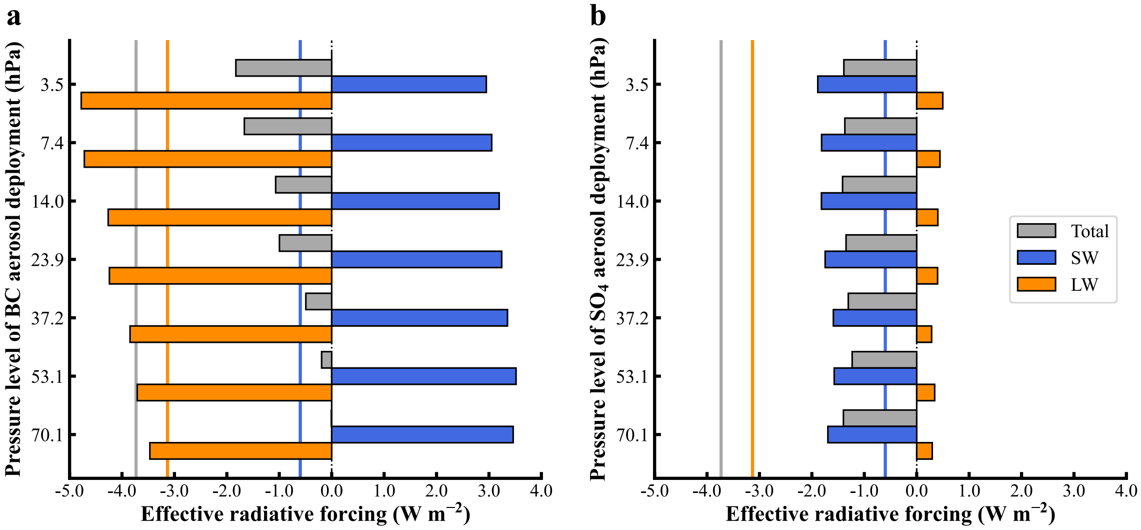

Supplementary Figure 3. Decompositions of effective radiative forcing of horizontal-uniformly prescribed (**a**) 0.5 Tg BC aerosols and (**b**) 5.0 Tg SO_4 aerosols at each of the highest 7 sigma levels of aerosol inputs, respectively, simulated by GFDL-AM2.5 model, into the shortwave and longwave components. These sigma levels are converted to approximate pressure levels by multiplying by 1000 hPa and are shown in the figure. The vertical lines represent the corresponding flipped components of doubling CO_2.

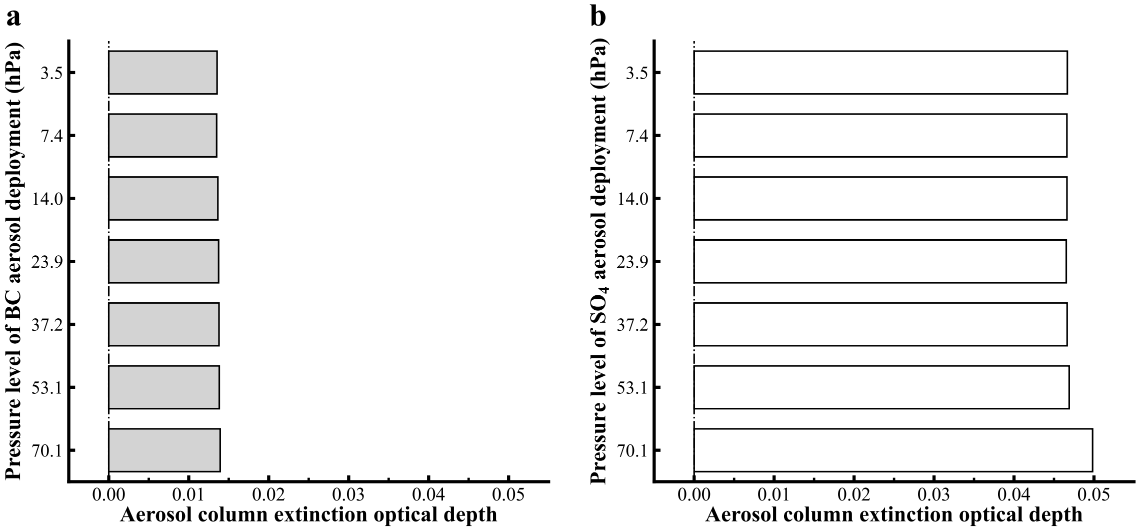

Supplementary Figure 4. Aerosol column extinction Optical Depth (AOD) of horizontal-uniformly prescribed (**a**) 0.5 Tg BC aerosols and (**b**) 5.0 Tg SO_4 aerosols separately at each of the highest 7 sigma levels of aerosol inputs, respectively, simulated by the GFDL-AM2.1 model. Results are shown only from GFDL-AM2.1, as AOD data were not saved for GFDL-AM2.5. Sigma levels are converted to approximate pressure levels by multiplying by 1000 hPa and presented in the figure. Filled and open bars represent AOD from 0.5 Tg BC and 5.0 Tg SO_4 aerosols, respectively, consistent with Supplementary Figure 2.

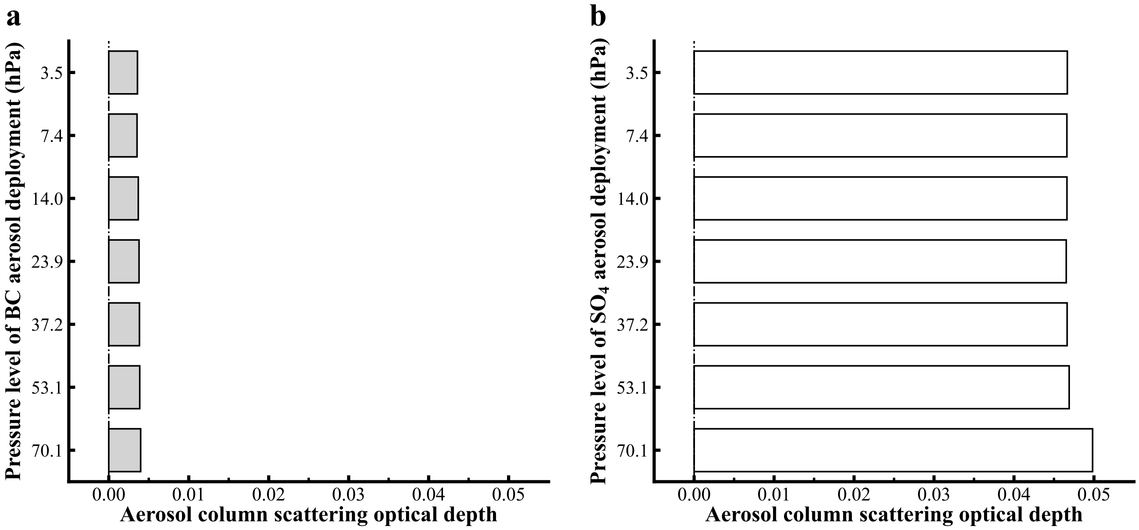

Supplementary Figure 5. Same as Supplementary Figure 4, except showing the scattering component of aerosol column optical depth.

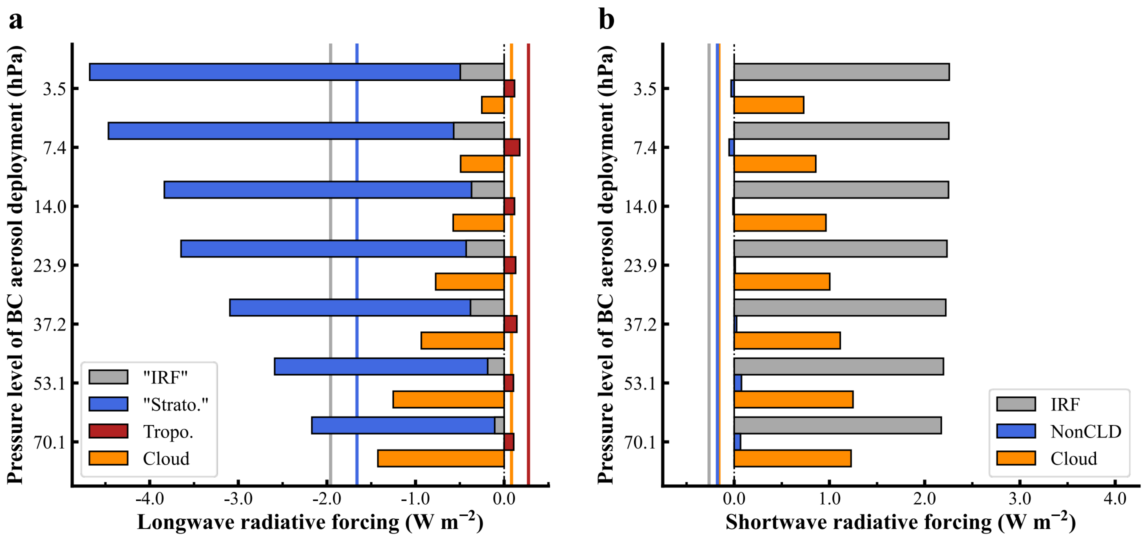

Supplementary Figure 6. Decomposition of (**a**) longwave and (**b**) shortwave effective radiative forcing of horizontal-uniformly prescribed 0.5 Tg BC aerosols at each of the highest 7 sigma levels of aerosol inputs, respectively, simulated by GFDL-AM2.5 model, into the instantaneous radiative forcing (IRF) and individual rapid adjustment components. Sigma levels are converted to approximate pressure levels by multiplying by 1000 hPa and presented in the figure. The solid vertical lines represent the corresponding flipped components of doubling CO_2.

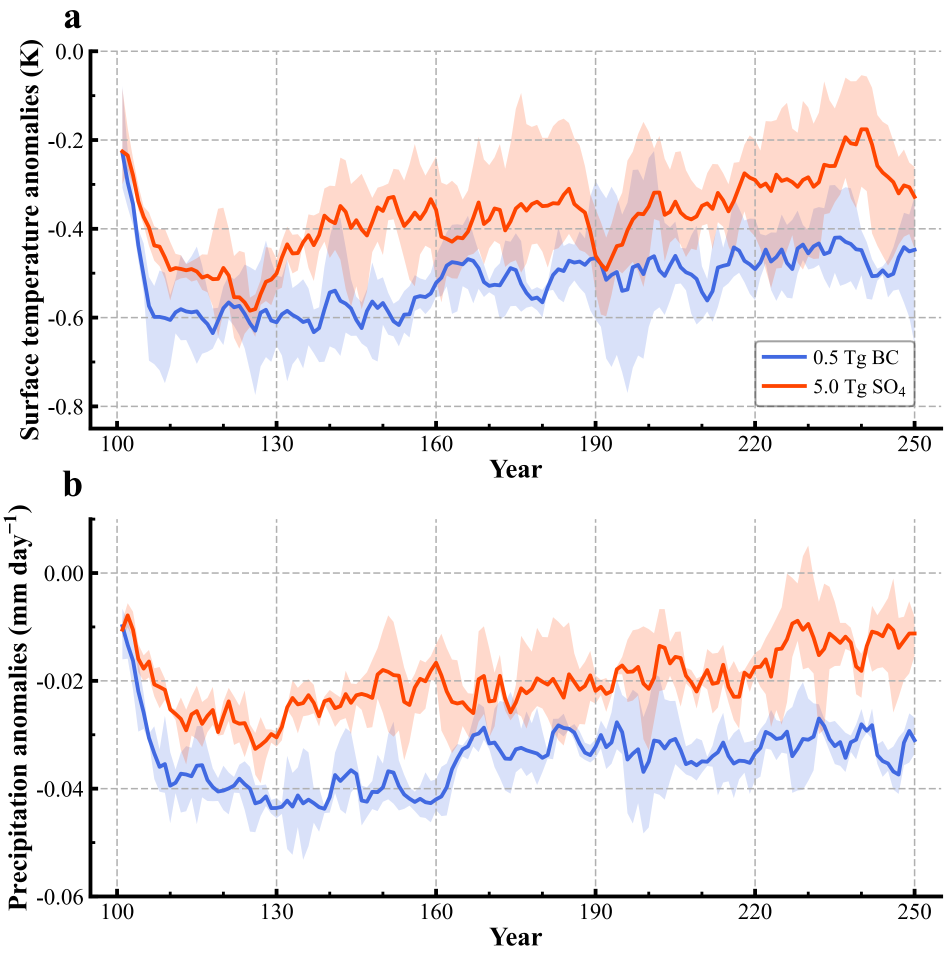

Supplementary Figure 7. Same as the solid lines and shading of Fig. 3, except showing climate intervention results starting from a non-equilibrium CO_2 doubling case simulated by GFDL-CM2.1. The non-equilibrium CO_2 doubling state refers to year 100 of the 1pct2×CO2 simulations, where CO_2 concentrations increase by 1% per year until doubling at year 70, then remain fixed for the remainder of the simulation. Anomalies are calculated relative to the climatological mean of a 30-year period centered on year 100 (years 85–115) of the 1pct2×CO2 simulations.

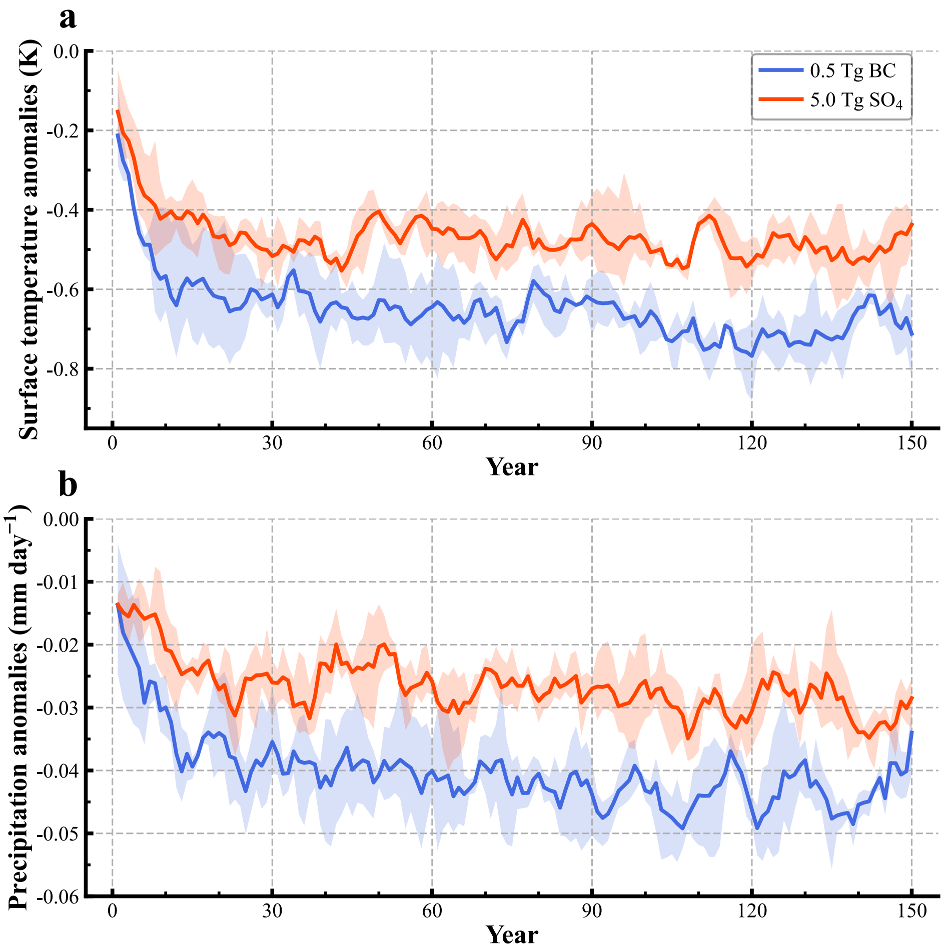

Supplementary Figure 8. Same as the solid lines and shading of Fig. 3, except showing climate intervention results starting from an equilibrium CO_2 doubling case simulated by GFDL-CM2.1. The equilibrium CO_2 doubling state refers to the later stages of long-run, abrupt CO_2 doubling simulations, where the atmosphere has reached equilibrium with no further warming or energy imbalance. Anomalies are calculated relative to the climatological mean of the equilibrium simulations.

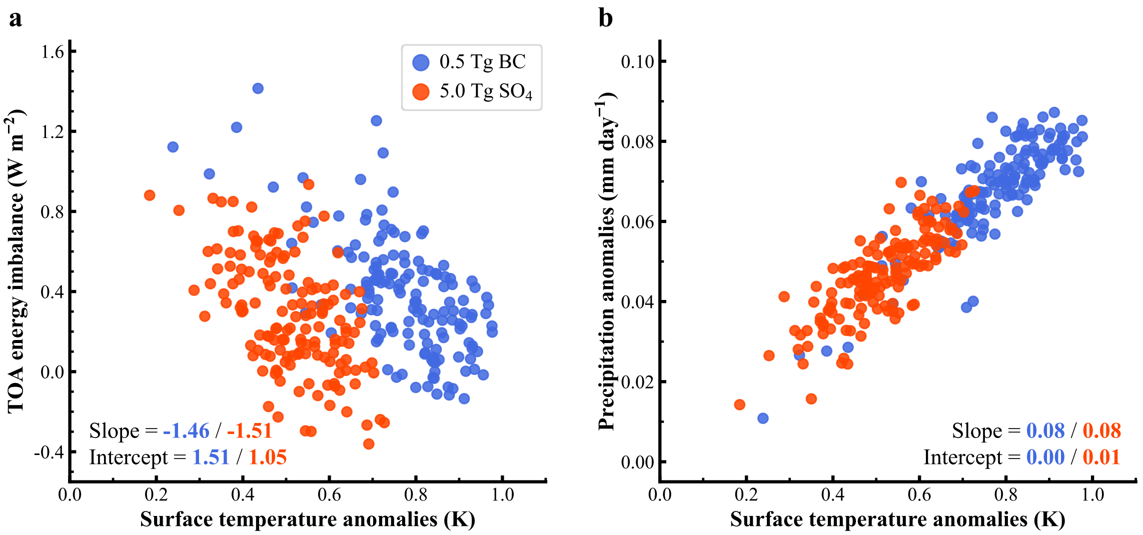

Supplementary Figure 9. Scatter plots of flipped ensemble-mean surface air temperature anomalies versus (**a**) flipped TOA energy imbalance and (**b**) flipped precipitation anomalies, for the GFDL-CM2.1 model simulations forced with 0.5 Tg BC and 5 Tg SO_4 aerosols separately, by horizontal-uniformly prescribing the targeted aerosols at the second highest sigma level of aerosol inputs, beginning from the pre-industrial control runs. The individual dots represent the ensemble mean of 3 members with different conditions taken from a pre-industrial control simulation by the GFDL-CM2.1 model. The anomalies are flipped to match the conventional Gregory plots, which are designed for global warming cases. Therefore, the y-intercepts noted in the plots need to be interpreted by reversing signs.

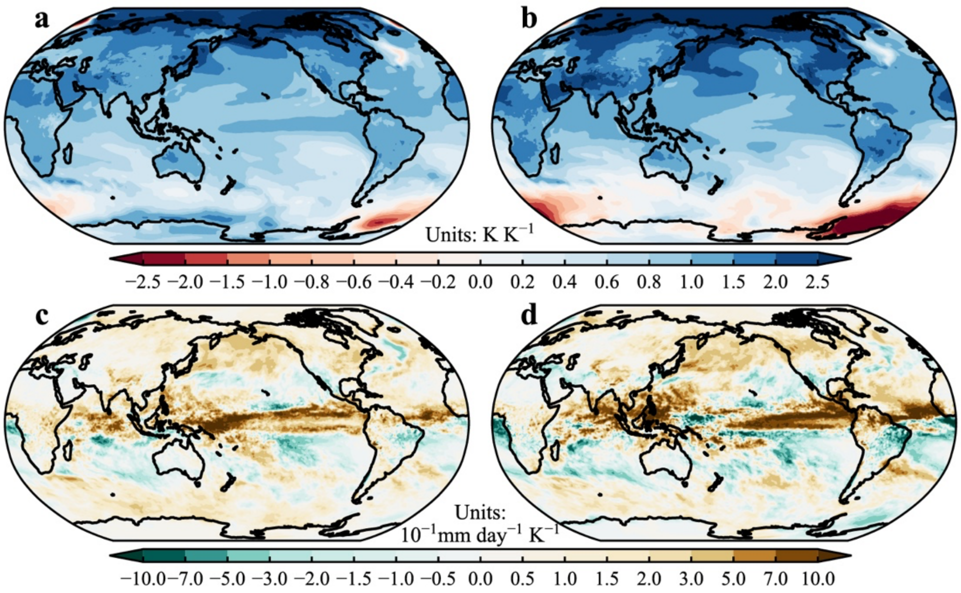

Supplementary Figure 10. Same as Fig. 4, except for the results of the single realization of the GFDL-CM2.5-FLOR model.

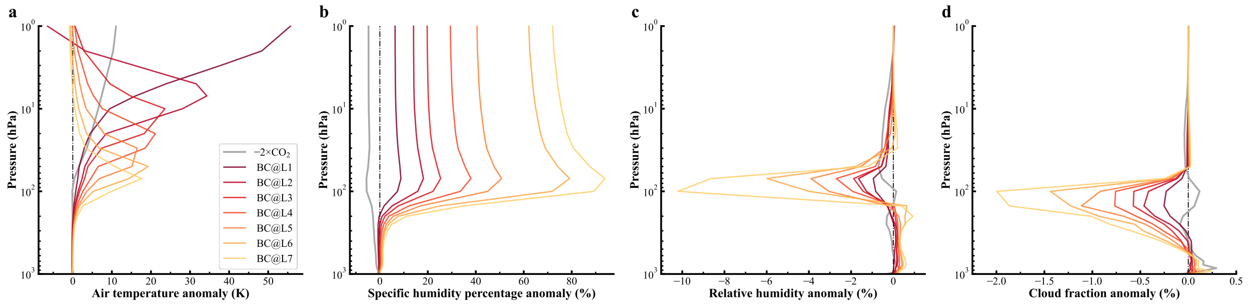

Supplementary Figure 11. Profiles of GFDL-AM2.5 simulated (**a**) air temperature, (**b**) specific humidity (shown as percentage anomalies), (**c**) relative humidity, and (**d**) cloud fraction anomalies in response to horizontal-uniformly prescribed 0.5 Tg BC aerosols at each of the highest 7 sigma levels of aerosol inputs [the level number is defined with direction from top to bottom (or altitude from high to low)], respectively. Since absolute changes in specific humidity are too small to visualize, anomalies are presented as percentages relative to the control simulation. Flipped profiles of air temperature, specific humidity, relative humidity, and cloud fraction responses to a doubling of CO_2 are also provided for reference.

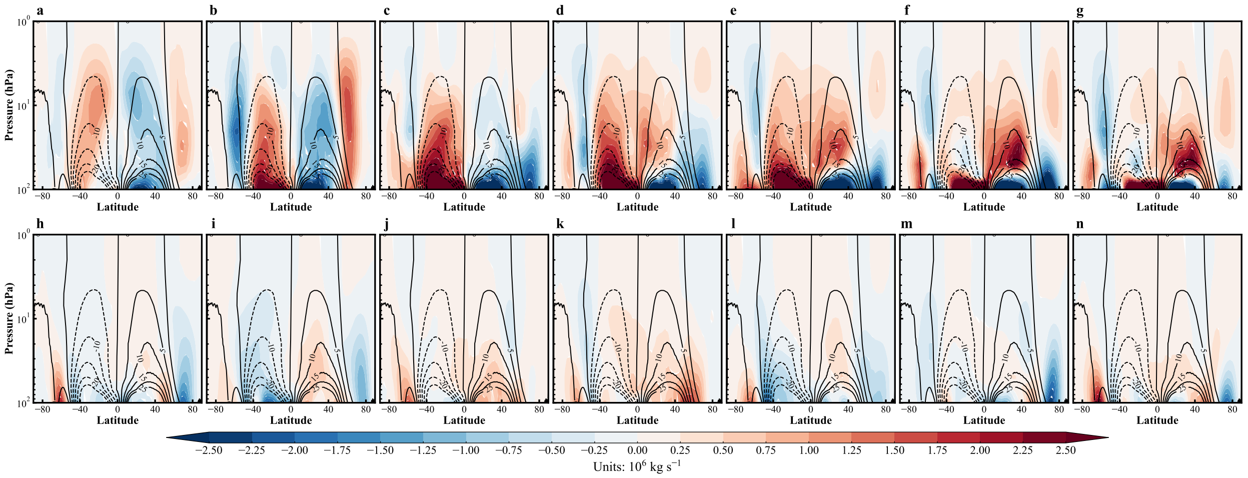

Supplementary Figure 12. Residual mass stream function anomalies (shading) in response to in response to horizontal-uniformly prescribed 0.5 Tg BC (**a–g**) and 5.0 Tg SO_4 (**h–n**) aerosols at each of the highest 7 sigma levels of aerosol inputs. From left to right, panels show results for aerosols placed progressively lower in the stratosphere. Contours in each subplot display the climatological distribution from the control simulation. The residual circulation is calculated following established formulations[1,2].

Supplementary References

1. Andrews, D. G., Holton, J. R. & Leovy, C. B. *Middle atmosphere dynamics* (Academic Press, 1987).
2. Salby, M. L. *Fundamentals of atmospheric physics* (Academic Press, 1996).
